# Relationship between Telomere Length, Genetic Traits and Environmental/Occupational Exposures in Bladder Cancer Risk by Structural Equation Modelling

**DOI:** 10.3390/ijerph15010005

**Published:** 2017-12-21

**Authors:** Sofia Pavanello, Angela Carta, Giuseppe Mastrangelo, Manuela Campisi, Cecilia Arici, Stefano Porru

**Affiliations:** 1 Department of Cardiac, Thoracic and Vascular Sciences, Unit of Occupational Medicine, University of Padova, 35128 Padova, Italy; giuseppe.mastrangelo@unipd.it (G.M.); manuela.campisi@studenti.unipd.it (M.C.); 2 Department of Medical and Surgical Specialties, Radiological Sciences and Public Health, Section of Public Health and Human Sciences, University of Brescia, 25123 Brescia, Italy; angela.carta@unibs.it (A.C.); cecilia.arici@unibs.it (C.A.); 3 University Research Center “Integrated Models for Prevention and Protection in Environmental and Occupational Health” (MISTRAL), University of Brescia, 25123 Brescia, Italy; stefano.porru@univr.it; 4 Department of Diagnostics and Public Health, Section of Occupational Health, University of Verona, 37134 Verona, Italy

**Keywords:** telomere length, environmental exposures, occupational exposures, DNA adduct, bladder carcinogenesis, cancer prevention, genetic polymorphisms, structural equation modelling, case-control study

## Abstract

*Background*: Telomere length (TL) maintenance plays an important role in bladder cancer (BC) and prognosis. However the manifold influence of everyday life exposures and genetic traits on leucocyte TL (LTL), is not fully elucidated. *Methods*: Within the framework of a hospital-based case (*n* = 96)/control (*n* = 94) study (all Caucasian males), we investigated the extent to which LTL and BC risk were modulated by genetic polymorphisms and environmental and occupational exposures. Data on lifetime smoking, alcohol and coffee drinking, dietary habits and occupational exposures, pointing to aromatic amines (AAs) and polycyclic aromatic hydrocarbons (PAHs) were collected. Structural equation modelling (SEM) analysis appraised this complex relationships. *Results*: The SEM analysis indicates negative direct links (*p* < 0.05) between LTL with age, DNA adducts, alcohol and NAT2, and positive ones with coffee, MPO and XRCC3; and between BC risk (*p* < 0.01) with cigarettes, cumulative exposure to AAs and coffee, while are negative with LTL and age. There was evidence of indirect effects (*p* < 0.05) on BC risk, probably via LTL reduction, by age and NAT2 (positive link), MPO and XRCC3 (negative link). Conclusions: Our study supports evidence that LTL attrition is a critical event in BC. The new finding that LTL erosion depends on some preventable everyday life exposures genetically modulated, opens new perspectives in BC prevention.

## 1. Introduction

Dysfunction in telomere maintenance plays an important role in human carcinogenesis; however, the complex relationship between telomere protection and the risk of developing bladder cancer (BC) has not been thoroughly studied. Telomeres are hexameric nucleotide (TTAGGG) n-protein repeats on the distal ends of eukaryotic chromosomes that are critical in maintaining the genome stability of the cells [1]. Telomeric repeats in normal somatic tissue shorten by 30 to 200 bp after each mitotic division eroding chromosomal terminations [2]. A specific enzyme, telomerase is involved in telomere synthesis after mitosis, but is active only in progenitor cells and in certain diseases [3]. Telomeres, therefore, progressively shorten with each division of somatic cells, and their length, measured in peripheral blood leukocytes (LTL), is considered an indicator of biological age [4]. Continuing attrition of telomeres causes genetic instability that can favour, over time, the typical diseases associated with aging, including cancer [3]. Telomere shortening is a function of oxidative stress and antioxidant defences [4]. Environmental (e.g., cigarette smoking) and occupational exposure to bladder carcinogens, directly, by damaging DNA, and indirectly, by favouring the cumulative effect of oxidative damages and the onset of chronic inflammation, might accelerate the physiological process of telomere erosion and, consequently, facilitate the onset of chronic degenerative pathologies, cancer included [5]. Furthermore, it has been documented an association between leukocyte telomere length (LTL) reduction and some known risk factors for BC, such as exposure to nitrosamines [6,7] and polycyclic aromatic hydrocarbons (PAHs) [8,9]. Some retrospective case-control studies associated shorter LTL in patients with BC [7,10,11]. Long LTL was instead strongly associated with an increased BC risk in Egyptians [12]. These conflicting results are hard to be explained from the biological viewpoint. Moreover, no study apparently estimated the complex interactions between LTL, multiple genetic polymorphisms, occupational and everyday life exposure to aromatic amines (AAs) and PAHs, even evaluated by DNA adduct estimation and BC risk. 

We previously assessed the interaction between occupational and environmental exposures with metabolic and DNA-repair polymorphisms on the risk of BC in a retrospective hospital based case-control study [13,14,15,16]. BC that is the fourth most common cancer in men and the 11th one in women, is a typical example of multifactorial disease, whose etiology is characterized by the interaction between environmental/occupational and genetic risk factors [17,18]. Tobacco smoking and occupational exposure are major risk factors attributable (about 50% and 4–20%, respectively) to BC via exposure to AAs and PAHs, which lead to increase oxidative stress and DNA damage [13]. In particular, occupational exposure to AAs (e.g., dyestuffs industry and rubber textile and printing) and to PAHs (e.g., metallurgy and metalworking sectors, drivers and exposed to diesel emissions) has long time been associated with an increased incidence and mortality for BC [18,19]. On the other hand, increasing evidence suggests a significant influence of genetic predisposition, including that related to metabolism and DNA damaging repair, on BC incidence [13,14,15] accounting for almost 20% of the BC risk. In particular, the application of genome-wide analyses (GWAS), beside confirming the involvement of genetic variants linked to detoxification pathways of bladder carcinogens (NAT2, UGT1A, GSTM1), has led to the identification of a number of variants, among which the most accredited are those involved in the telomere’s biology, conferring an increased BC risk [20].

The aim of the present study is twofold: to investigate the extent to which LTL and BC risk were modulated by genetic polymorphisms, and environmental/occupational exposures, where exposure was also evaluated by leucocytes DNA adducts analysis; to explore whether LTL involved an additional increase in BC risk. These complex interrelationships have been appraised using the analysis of structural equation modelling (SEM).

## 2. Materials and Methods 

### 2.1. Subjects

The present study includes study population stemming from an earlier hospital-based case-control study fully described in previous publications [13,14,15]. Inclusion criteria were being male, aged 20–80 and Italian. In all the cases involved 199 newly diagnosed, histologically confirmed BC patients, admitted to the urology departments of two large hospitals from 1997 to 2000. Controls were a total of 214 non-neoplastic urological patients matched to cases by age (±5 years), period and hospital of admission. Exposure to occupational and environmental carcinogens was evaluated by a standardized questionnaire (cumulative exposure to AA and PAHs) and by level of DNA adducts in leucocytes (dose to the target). A written informed consent was obtained from each subject; the local Ethical Committee approved the study (protocol number 2859/9185, 4 September 1996). In the present study, we had considered 96 cases and 94 controls for which was still available a sufficient DNA aliquot for the analysis of telomeres.

#### 2.1.1. Data Collection

A trained interviewer collected information on demographic variables, lifetime smoking history, coffee and other liquid consumption, dietary habits, lifetime occupation history by questionnaire. Occupational exposures to PAHs and AAs were estimated according to methodology described in previous publication [13,14,15]. An index of cumulative exposure to AAs and PAHs, separately, was calculated as product (*i × f × l*) of length (*l*), intensity (*i*) and frequency (*f*) of exposure in each job, summing up as many products as were necessary to take into account all jobs done. Life-long consumption of cigarettes was calculated as pack-years. The lifelong time-weighted average of cups/day of coffee was recoded as 0 (never drinkers), ≤3, 4, ≥5 cups/day. PAHs containing food, fruit, large leaf vegetables and other vegetables consumption was divided into four categories (less than once/month; less than once/week; 1–3 times/week; more than 3 times/week). Job titles and individual activities, as well as occupational exposures to AAs, were blindly coded by an occupational physician according to methodology described in previous publication [13,14,15]. Occupations involving exposure to AAs were attributed to 11 International Standard Classification of Occupations codes for job tasks (1-61.30: Painter, Artist; 3-70.20: Mail Sorting Clerk; 5-70.30: Barber-Hairdresser; 7-41.40: Mixing- and Blending-Machine Operator, Chemical and Related Processes; 8-01.10: Shoemaker, General; 8-11.20: Cabinetmaker; 8-73.70: Vehicle Sheet-Metal Worker; 9-01.35: Rubber Moulding-Press Operator; 9-31.20: Building Painter; 9-39.20: Brush-Painter, except Construction; 9-39.30: Spray-Painter, except Construction) and 11 International Standard Industrial Classification of all Economic Activities codes for industrial activities (3240: Manufacture of footwear, except vulcanized or moulded rubber or plastic footwear; 3320: Manufacture of furniture and fixtures, except primarily of metal; 3521: Manufacture of paints, varnishes and lacquers; 3559: Manufacture of rubber products not elsewhere classified; 3819: Manufacture of fabricated metal products except machinery and equipment not elsewhere classified; 3824: Manufacture of special industrial machinery and equipment except metal- and wood-working machinery; 3843: Manufacture of motor vehicles; 5000: Construction; 9415: Authors, music composers and other independent artists not elsewhere classified; 9513: Repair of motor vehicles and motorcycles; 9591: Barber and beauty shops).

#### 2.1.2. Analysis on DNA from Peripheral Blood Leucocytes

Blood samples were collected from all the subjects during hospital admission and on the same day processed by centrifugation for obtaining peripheral blood leucocytes. The protocol for automated DNA extraction was performed according to Extragen kit (Extragen, by ELITech Group S.p.A., Torino, Italy) following the manufacturer’s instructions as previously described [13,14,15,16]. In particular 2.5 mL of buffy coats prepared from up to 10 mL of whole blood were processed for DNA extractions. A typical yield ranged from 150 to 400 µg DNA/extraction from a normal donor.

#### 2.1.3. 32P-Post-Labeling Analysis of DNA Adducts

Aliquots of 5 μg DNA were assayed for the presence of aromatic-DNA adducts by 32P-postlabeling after enrichment with Nuclease P1 as previously described [21]. Resolution of DNA adducts was performed by multidirectional thin-layer chromatography (TLC), using polyethyleneimine (PEI)-cellulose plates [21]. DNA were enzymatically digested to 3’-mononucleotides with 0.14 U/µg DNA of micrococcal nuclease and 1 mU/µg DNA of spleen phosphodiesterase for 3–4 h at 37 °C. After the enrichment procedure by Nuclease P1 digestion, DNA bases were labelled with 50 µCi of [gamma-32P] ATP with a specific activity of 5000 Ci per mmol by using 2.5 units of T4 polynucleotide kinase. 20 µL of postlabeled sample were spotted on the origin of a premarked PEI cellulose sheet and run for the multidirectional TLC chromatography. DNA adducts levels were measured as relative adduct level per 10^8^ nucleotides.

#### 2.1.4. Genotyping

Genotyping of glutathione S-transferase M1 (GSTM1) null, GSTT1 null, GSTP1 I105V, *N*-acetyltransferase 1 (NAT1) fast, NAT2 slow, cytochrome P450 1B1 (CYP1B1) V432L, sulfotransferase 1A1 (SULT1A1) R213H, myeloperoxidase (MPO) G-463A, catechol-*O*-methyltransferase (COMT) V108M, manganese superoxide dismutase (MnSOD) A-9V, NAD(P)H:quinone oxidoreductase (NQO1) P187S, X-ray repair cross-complementing group 1 (XRCC1) R399Q, XRCC3 T241M, and xeroderma pigmentosum complementation group (XPD) K751Q polymorphisms was assessed using Amplification Refractory Mutation System assay [13,14,15]. 

#### 2.1.5. Leucocytes Telomere Length Analysis

Leucocytes telomere length (LTL) measured by the real-time method (RT-PCR) following the Cawthon procedure [22] as we previously used [9]. Briefly, after DNA extraction from leukocytes, LTL was determined by the ratio between the number of repeated copies of the telomere (T), compared to a single-copy gene known (S), (T/S ratio) [9]. The single copy gene used in this study was human b-globin (HBG). The analysis was conducted using the RT-PCR system StepOne plus equipment (Life Technologies, Applied Biosystems, Milan, Italy). In each series of analysis it has been inserted a standard calibration curve made up of six points and generated by a pool of DNA diluted in series, the concentration of which varies between 40 ng and 1.25 ng. All samples were examined in triplicate and the average of the three T/S ratios was used in the statistical analysis. To evaluate the reproducibility of the ratio T/S, the test was repeated for the 10% of the samples on different days. The coefficient of variation in the analysis was 3.0% [16].

### 2.2. Statistical Analysis 

The test of Wilcoxon-Mann-Whitney was used to compare the key characteristics of cases and control, that include age, years of school, tobacco smoke, coffee and/or alcohol consumption, body mass index (BMI), vegetables consumption, cumulative exposure to AA and PAHs, levels of DNA adducts and LTL.

Structural equation modeling (SEM) analysis was used to appraise the complex relationships among the variables. In fitting SEM, age at diagnosis, lifelong consumption of cigarettes (pack-years), coffee consumption and alcohol intake (cumulative consumption), myeloperoxidase (MPO), cumulative exposure to aromatic amines—variables associated with BC risk according to previous publications—plus DNA adducts (transformed in logarithm), X-ray repair cross-complementing protein 1 and 3 (XRCC1 and XRCC3), *N*-Acetyl Transferase 2 (NAT2), manganese superoxide dismutase (MnSOD) and cytochrome P450 1B1 (CYP1B1) were used as exogenous variables (corresponding to predictors in regression based techniques). BC risk and LTL (expressed as square root to normalize the distribution) were the endogenous variables (corresponding to outcome variables). There were two alternative hypotheses which stated: each endogenous variable could be affected by one or more exogenous variables (hypothesis 1); BC risk could also be influenced indirectly through the variable “LTL” (hypothesis 2). The two competing hypotheses were converted in two models of structural equations, to find which model fitted best the observed data. SEM structural equations were fitted with “asymptotic distribution free” method because it did not make assumption on joint normality of all the variables. The effect of each exogenous variable was expressed as standardized (or beta) coefficients that make comparisons easily by ignoring the independent variable’s scale of units. Both direct and indirect effects were estimated by SEM. SEM results were both tabulated and presented graphically. We used two SEM’s goodness-of-fit statistics: (1) the chi square test for “model versus saturated” (the saturated model is the model that fits the covariances perfectly); and (2) the stability index obtained from the analysis of simultaneous equation systems. The analysis was carried out with the statistical package STATA 13 (StataCorp., College Station, TX, USA).

The sample size required for SEM is dependent on model complexity, the estimation method used, and the distributional characteristics of observed variables [23]. The best option is to consider the model complexity (i.e., the number of exogenous variables) and the following rules of thumb: minimum ratio 5:1 [23,24]; recommended ratio 10:1 [25,26]; recommended ratio 15:1 for data with no normal distribution [25]. With 12 exogenous variables used in the SEM model, we should have 180 (= 15 × 12) subjects but they were actually 190, fulfilling the above requirements. The power should therefore be >0.80.

## 3. Results

Table 1 shows that the main characteristics of 96 cases and 94 controls were similar, except for pack-years (significantly higher in cases) and LTL (significantly higher in controls).

Table 2 shows the beta coefficients (“minus” sign indicating an inverse relationship) with 95% confidence intervals and *p*-values for two structural equation models estimated by SEM. LTL and BC risk were the endogenous variables for the first and second model, while the exogenous variables were the same for both models, respectively. Both direct and indirect effects are shown.

1. Direct effects. The first model shows that LTL is negatively associated with age (*p* = 0.000), DNA adducts (*p* = 0.017), alcohol intake (*p* = 0.017) and NAT2 (*p* = 0.018), and positively association with coffee (*p* = 0.016), MPO (*p* = 0.009) and XRCC3 (*p* = 0.004). The second model shows that BC risk significantly increased with consumption of cigarettes (*p* = 0.000), cumulative exposure to AAs (*p* = 0.003) and coffee (*p* = 0.006), while it decreased with LTL (*p* = 001) and age (*p* = 0.019).
2. Indirect effects. The first model shows no indirect effects. The second model shows that, via LTL reduction, BC risk increased with age (*p* = 0.007) and NAT2 (*p* = 0.011), while it decreased with MPO (*p* = 0.029) and XRCC3 (*p* = 0.003).

These findings supported the hypothesis that BC risk in our population was increased directly by LTL reduction, and was further affected through shortened LTL by several other variables, such as age and polymorphisms in NAT2, XRCC3, and MPO, that did not have a direct effect on BC risk. 

Concerning the goodness-of-fit statistic, the chi square test was 0.00 (*p* = 1.00) indicating no difference against a saturated model, and the stability index was 0.0, signifying that SEM model satisfied stability condition.

**Figure 1:**
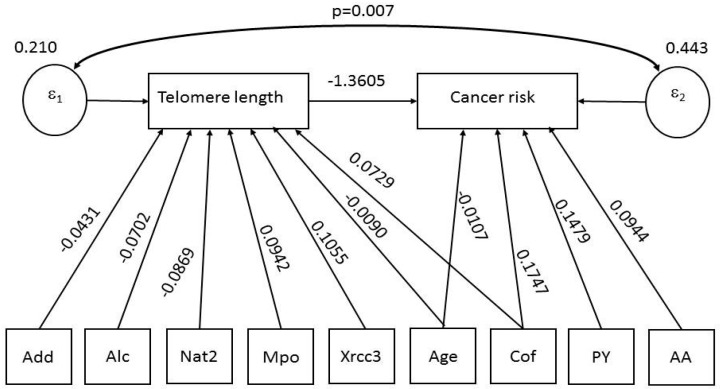
Path diagram of results shown in Table 2: variables (square boxes); causal flow (arrows); and paths (arrowed route); error terms for each equation (circles) and correlation between errors (curved path) with the corresponding p-value. The estimated beta coefficients appeared along the paths. Figure legend: Age = Age at diagnosis; Mpo = Mieloperoxidase; PY = Pack-years; Add = DNA adducts (ln); Cof = Coffee, (cumulative); AA = Aromatic amines (cumulative); Alc = Alcohol (cumulative); XRCC3 = X-ray repair cross-complementing protein 3; Nat2 = *N*-acetyl transferase 2.

Using the graphical interface of SEM, the same results (only direct effects) shown in Table 2 were displayed as path diagram in Figure 1. In this figure, square boxes stand for variables, arrows specify the direction of causal flow, an arrowed route is a path, and the estimated beta coefficients appeared along the paths. The “error term” for each equation is represented by a circle, and the correlation between errors is displayed as a curved path. It can be seen that error1 and error2 have a contemporaneous cross-equation correlation (*p* = 0.007). Therefore, the two equations were related through the correlation in their errors. 

## 4. Discussion

In this paper we report direct negative links between LTL with age, DNA adducts, alcohol and NAT2, and positive (protection) ones with coffee, MPO and XRCC3; and between BC risk with cigarettes, cumulative exposure to AAs and coffee, while are negative with LTL and age. There was evidence of indirect effects on BC risk, via LTL reduction, by age and NAT2, MPO and XRCC3 (negative link).

The negative relationship between LTL and DNA adducts, is in line with our previous findings in coke over workers highly exposed to occupational PAHs carcinogens [9], and would suggest that adduct formation may perhaps have a direct role in shortening LTL. DNA adducts, in fact, such as those we determined by P32 post-labelling are the results of the stereoselective binding of polyromantic compounds, AA included, to the exocyclic N2 of guanine nucleotides, that are considered the primary essential damaging event in bladder carcinogenicity [27]. In particular telomeres, as triple-G-containing sequences, may represent a sensitive target for damage by such AA genotoxic compounds. Double-strand breaks and interference with replication fork generated by the bulky-damaged telomeric bases may directly induce telomere shortening [28]. Then AA-adduct formation and the consequent telomere attrition may be modulated by a decrease in AA detoxification due to the specific NAT2 slow polymorphism. Furthermore, the formation of adduct in the proteins of the telomere-sheltering complexes, NAT2-modulated too, could be also considered as an alternative event accounting for shorter LTL, as an additional mechanism. 

LTL is found also to be modulated by some other genetic polymorphisms such as MPOA and XRCC1399Arg. The genetic polymorphism of enzymes involved in individual response to oxidative stress (MPO) and repair (XRCC3) is likely involved in modulating the individual response to environmental exposures such as tobacco smoking, coffee drinks, AAs exposure and DNA adducts formation too [13,21,29]. In particular, on one hand MPOA allele is associated with a reduced mRNA expression that in turn may shrunk its action on procarcinogen activation of tobacco smoke carcinogens [30], while XRCC1399Arg polymorphism, that presents higher DNA repair activity [15,29], was previously associated with lower levels of bulky DNA adducts [31,32]. In the present study LTL are longer in carriers MPOA and XRCC1399Arg, confirming the protective role of MPOA and XRCC3 even on telomere stability. 

The significance of the studied polymorphisms is related to the fact that the studied variants are those of enzymes that participate to the metabolic and DNA repair pathways of bladder carcinogens such as AAs and PAHs, and these are relevant functional variants. 

The literature findings regarding age and LTL is also supported giving a qualitative meaning to our results. The relationship of LTL with alcohol drinking is in line with our previous findings on shorter LTL in abusers with higher alcohol intake [33] confirming alcohol drinking as an important biological aging factor.

We found a positive (protective) effect of coffee consumption on LTL. Although the associations between coffee intake, disease, and mortality have been investigated multiple times, research evaluating the relationship between caffeine intake and telomere length relationship is extremely rare. Our results is in line with a previous study by Liu et al. [34] that showed a significant association between longer telomeres and caffeinated coffee consumption in 4780 women. While previous studies on coffee consumption and telomere length have provided inconsistent findings [35,36] because did not exclude decaffeinated coffee consumption. Present findings and those by Liu et al. [34] would suggest that some specific compounds contained in coffee may protect DNA integrity. In fact intervention studies have shown that coffee consumption reduces spontaneous DNA strand breaks [37] and protects against chemical-induced DNA damage [38,39] and oxidative DNA damage [40]. These intervention studies had study periods ranging from 3 days [39] to 4 weeks [37] and coffee consumption amount ranging from 600 mL/day [38] to 1 L/day [39]. In addition animal and cell line studies have shown that specific compounds in coffee such as chlorogenic acid [40,41] and diterpenes [42,43] may protect against DNA damage. 

With regard to BC risk, the literature is scanty on the relationship with occupational exposures to AAs and PAHs, and DNA adducts; those few published studies have only found an exposure-independent association between adducts and BC risk [44]. Our study precisely evaluated the occupational exposure history to AAs and PAHs, and noted a significant correlation between occupational exposure to AAs and BC (direct effect). Therefore occupational exposure to AAs is confirmed as central risk factor for BC development. In addition, the correlation we found between AAs exposure and BC risk is biologically plausible, because AAs are activated in liver and transported by blood proteins to the bladder where, under acidic conditions [45] or, enzymatically by O-acetylation of *N*-hydroxyarylamine (predominantly by the *N*-acetyltransferase 1 (NAT1) isozyme), are further activated to the ultimate carcinogen [46]. Unlike AAs, cumulative exposure to PAHs was not associated with BC risk in our study population. Experimental evidence suggests that PAHs are slowly absorbed through most tissues. For instance, in the case of dermal exposure, considered the main route in the industry [47,48], absorption accounts for a small fraction of applied dose, and PAHs are enzymatically activated and degraded at this site of entry [49,50,51]. The concentration and persistence of PAHs in the lung is largely related with inhalation of PAHs containing dust [51,52]. The high propensity of PAHs to act as carcinogens at the sites of entry is supported by several experimental studies [53].

There was evidence of indirect effects on BC risk, probably mediated by LTL reduction, of age and NAT2 (positive link), MPO and XRCC3 (negative link). The latter associations have been previously reported [13,14,15,16]; this study adds that these relations are indirect effects likely mediated by LTL reduction. 

As described by Cawthon and colleagues in a study on an elderly population [54], LTL shortening, considered a hallmark of cellular aging, is associated with an increase in mortality rate. Recently, some studies have coherently shown lower survival and lower LTL, in patients with different kinds of tumours, including BC [11,54,55,56]. Therefore, our results seem to be suggestive that patients with BC who are older and with shorter LTL could be at poorer prognosis. This aspect is warranted to be prospectively scrutinized. Further studies in larger populations should investigate the complex interrelationships among LTL, environmental and occupational variables as well as genetic endpoints, especially taking into account the relevant clinical and prognostic parameters along the course of BC. 

## 5. Conclusions

In conclusion, the new relevant findings are that LTL erosion associates directly and directly with BC risk, strengthening the evidence of a central role of LTL in bladder carcinogenesis. Moreover, at the moment of BC diagnosis, LTL was found to decrease with some genetic polymorphisms and higher levels of DNA adduct and alcohol intake and to increase with coffee consumption. 

## Figures and Tables

**Table 1:** Characteristics of the study population.

Variables	Cases (*N* = 96)		Controls (*N* = 94)		Mann-Withney
	Average ± SD	*N* (%)	Average ± SD	*N* (%)	*p*
Age (years)	61.5 ± 10.9	96 (100)	60.3 ± 11.7	94 (100)	0.6224
Years of school	7.41 ± 3.35	95 (99)	8.38 ± 3.67	93 (99)	0.0530
Tobacco smoke (pack-years)	32.8 ± 20.6	96 (100)	23.2 ± 20.6	94 (100)	0.0008
coffee consumption (coffee-years)	2.76 ± 2.67	96 (100)	2.05 ± 1.49	94 (100)	0.1158
Alcohol consumption (alcohol-years)	93.4 ± 104	96 (100)	107 ± 117	94 (100)	0.4154
BMI (Kg/m^2^)	26.0 ± 3.61	85 (86)	25.9 ± 3.20	88 (94)	0.8329
Vegetables consumption (vegetables/week)	2.43 ± 0.80	96 (100)	2.52 ± 0.60	94 (100)	0.8142
Cumulative exposure to AA	79.7 ± 78.0	11 (11)	18.6 ± 17.5	6 (6)	0.1880
Cumulative exposure to PAHs	46.5 ± 39.1	37 (38)	31.5 ± 31.6	35 (37)	0.4922
DNA adducts (ln)	1.11 ± 1.31	96 (100)	0.84 ± 1.11	94 (100)	0.1831
LTL (T/S)	1.55 ± 1.14	96 (100)	2.03 ± 1.42	94 (100)	0.0123

SD: standard deviation; BMI: body mass index; AA: aromatic amines; PAH: polycyclic aromatic hydrocarbons; LTL: leucocytes telomere length.

**Table 2:** SEM results (beta coefficients, 95% confidence intervals and *p*-values) for endogenous variables of structural equations: direct and indirect effects.

Endogenous Variables	Exogenous Variables	Direct Effects			Indirect Effects		
		Beta Coefficient	95% Confidence Interval	*p*-Value	Beta Coefficient	95% Confidence Interval	*p*-Value
**LTL (RQ)**	**Age**	**−0.0090**	**−0.0132**; **−0.0049**	**0.000**	0		
	**DNA Adducts (ln)**	**−0.0431**	**−0.0787**; **−0.0075**	**0.017**	0		
	**Alc**	**−0.0702**	**−0.1278**; **−0.0127**	**0.017**	0		
	PY	0.0385	−0.0107; 0.0879	0.125	0		
	**Cof**	**0.0729**	**0.0138**; **0.1320**	**0.016**	0		
	AA	−0.0240	−0.0865; 0.0383	0.450	0		
	**Nat2**	**−0.0869**	**−0.1591**; **−0.0147**	**0.018**	0		
	**MPO**	**0.0942**	**0.0238**; **0.1647**	**0.009**	0		
	**XRCC3**	**0.1055**	**0.0333**; **0.1777**	**0.004**	0		
	XRCC1	0.0335	−0.0360; 0.1031	0.345	0		
	MnSOD	−0.0014	−0.1202; 0.1174	0.981	0		
	CYP1B1	0.0098	−0.0673; 0.0870	0.803	0		
**BC risk**	**LTL (RQ)**	**−1.3605**	**−2.1939**; **−0.5271**	**0.001**	0		
	**Age**	**−0.0107**	**−0.0198**; **−0.0017**	**0.019**	**0.0123**	**0.0034**; **0.0213**	**0.007**
	DNA Adducts (ln)	−0.0439	−0.1127; 0.0249	0.212	0.0587	−0.0033; 0.1207	0.064
	Alc	−0.0663	−0.1761; 0.0434	0.236	0.0956	−0.0081; 0.1992	0.071
	**PY**	**0.1478**	**0.0726**; **0.2231**	**0.000**	−0.0525	−0.1283; 0.0233	0.174
	**Cof**	**0.1747**	**0.0505**; **0.2989**	**0.006**	−0.0992	−0.2058; 0.0073	0.068
	**AA**	**0.0944**	**0.0314**; **0.1575**	**0.003**	0.0328	−0.0517; 0.1172	0.447
	**Nat2**	0			**0.1183**	**0.0275**; **0.2090**	**0.011**
	**MPO**	0			**−0.1283**	**−0.2438**; **−0.0128**	**0.029**
	**XRCC3**	0			**−0.1436**	**−0.2385**; **−0.0487**	**0.003**
	XRCC1	0			−0.0456	−0.1379; 0.0466	0.332
	MnSOD	0.0904	−0.0757; 0.2566	0.286	0.0019	−0.1600; 0.1638	0.982
	CYP1B1	0			−0.0134	−0.1186; 0.0918	0.803

Legend: AA = aromatic amines (cumulative); Age = age at diagnosis; Alc = alcohol (cumulative); BC risk = bladder cancer risk; Cof = coffee (cumulative); CYP1B1 = cytochrome P450 1B1; DNA Adducts (ln); XRCC1 = X-ray repair cross-complementing protein 1; XRCC3 = X-ray repair cross-complementing protein 3; LTL (RQ) = leucocytes telomere length (expressed as square root); MnSOD = manganese superoxide dismutase; MPO = mieloperoxidase; Nat2 = *N*-acetyl transferase 2; PY = pack-years. Bold = statistically significant results.
